# A Taxonomy of Functional Upper Extremity Motion

**DOI:** 10.3389/fneur.2019.00857

**Published:** 2019-08-20

**Authors:** Heidi M. Schambra, Avinash Parnandi, Natasha G. Pandit, Jasim Uddin, Audre Wirtanen, Dawn M. Nilsen

**Affiliations:** ^1^Mobilis Lab, Department of Neurology, New York University School of Medicine, New York, NY, United States; ^2^Department of Rehabilitation Medicine, New York University School of Medicine, New York, NY, United States; ^3^Department of Neurology, Columbia University, New York, NY, United States; ^4^Department of Rehabilitation and Regenerative Medicine, Columbia University, New York, NY, United States

**Keywords:** primitive, rehabilitation, dose, upper extremity, function, stroke

## Abstract

**Background:** Functional upper extremity (UE) motion enables humans to execute activities of daily living (ADLs). There currently exists no universal language to systematically characterize this type of motion or its fundamental building blocks, called functional primitives. Without a standardized classification approach, pooling mechanistic knowledge and unpacking rehabilitation content will remain challenging.

**Methods:** We created a taxonomy to characterize functional UE motions occurring during ADLs, classifying them by motion presence, temporal cyclicity, upper body effector, and contact type. We identified five functional primitives by their phenotype and purpose: *reach, reposition, transport, stabilize*, and *idle*. The taxonomy was assessed for its validity and interrater reliability in right-paretic chronic stroke patients performing a selection of ADL tasks. We applied the taxonomy to identify the primitive content and motion characteristics of these tasks, and to evaluate the influence of impairment level on these outcomes.

**Results:** The taxonomy could account for all motions in the sampled activities. Interrater reliability was high for primitive identification (Cohen's kappa = 0.95–0.99). Using the taxonomy, the ADL tasks were found to be composed primarily of *transport* and *stabilize* primitives mainly executed with discrete, proximal motions. Compared to mildly impaired patients, moderately impaired patients used more repeated *reaches* and axial-proximal UE motion to execute the tasks.

**Conclusions:** The proposed taxonomy yields objective, quantitative data on human functional UE motion. This new method could facilitate the decomposition and quantification of UE rehabilitation, the characterization of functional abnormality after stroke, and the mechanistic examination of shared behavior in motor studies.

## Introduction

Upper extremity (UE) motor recovery after stroke is often incomplete in humans, attributed in part to insufficient rehabilitation training in the early months after stroke (1). In preclinical models, promotion of motor recovery is achieved with large doses of functional movement delivered early after stroke (2, 3). In humans, the optimal subacute rehabilitation dose to promote recovery is not known. Clinical research seldom identifies or quantifies the content of rehabilitation training (4), and the quantitative dosing of functional training has only been undertaken in chronic stroke (5). Having a language to systematically decompose rehabilitation into measurable units is an important first step toward optimizing rehabilitation delivery.

Functional UE movements enable engagement with the environment in a purposeful manner (6), and are a focus of rehabilitation training (7). Although functional movements are diverse given the many objects and goals encountered in daily living, they consist of a limited array of building-block motions called functional primitives (8, 9). Like words, functional primitives can be strung together to create a functional movement (akin to a sentence), which in turn are strung together to create an activity [akin to a paragraph; (10)]. Primitives have a motion phenotype that is surprisingly consistent even across species (11), indicating that their identification should be possible regardless of individual or activity.

There currently exists no universal vocabulary to systematically characterize functional UE primitives. Previous classification schemas have focused on identifying hand grasp and manipulation types (12, 13), single-joint motions (14), UE motions in dance (15), or motion types inferred from a cursor path (8). No approach to date has methodically defined and organized functional UE motion into a structured, rational framework.

We thus sought to create a taxonomy that characterizes functional UE motion by its fundamental primitives and their overarching motion features. We took a rank-based classification approach, grouping motions into classes by the similarity of their appearance, as observable by humans. We applied the taxonomy to functional activities performed by stroke patients, validating that the taxonomy could account for all constituent motions. We also confirmed a classification branch point, the phenotypic divergence between two classes, using machine learning. We furthermore found high inter-rater reliability for classification of motions in stroke patients. We finally applied the taxonomy to inspect the primitive content and motion characteristics of different ADLs, and to evaluate functional strategies made by stroke patients with different impairment levels.

## Methods

### Definitions

We first define terms pertaining to the functional primitive taxonomy, extending previous work (6, 12, 16). The taxonomy focuses on motion made in the context of ADLs, and definitions are made within this functional framework.

- Motion: generic displacement of a body part in space, agnostic to goal or function. We operationalize motion of the UE as the translation of the UE in space relative to a body coordinate frame centered at the pelvis (12).
- Functional motion: generic motion (or minimal-motion, **Figure 2**) that is goal-directed and volitional. Functional motion is made with respect to an object or target during task execution. This is a general term, irrespective of time scale or goals (see functional motion hierarchy, below).
- Workspace: general location of the functional motion, typically found between shoulder and waist (17).
- Object: entity that is the target of engagement by functional motion. In ADLs, this typically implies a hand-sized entity, but can also include a target on the surface of a larger entity (e.g., a part of the body, clothing) or a more diffuse substance (e.g., water, blowing air). Objects can be virtual (e.g., smartphone apps, virtual reality targets). Not all entities in proximity to the body are targets of engagement. For example, a table is not typically the target, but it supports other objects that are. Similarly, outside of the activity of dressing, clothing that is worn is typically not the target of engagement. During functional activities, a grasped object is often used to act on an object in the workspace (e.g., a fork to spear food). The taxonomy focuses solely on the motion/minimal-motion related to the grasped object, not its effect on a secondary object. Often, the motion for both the grasped and acted-on object is the same (e.g., transporting a fork to transport food).

**Figure 1:**
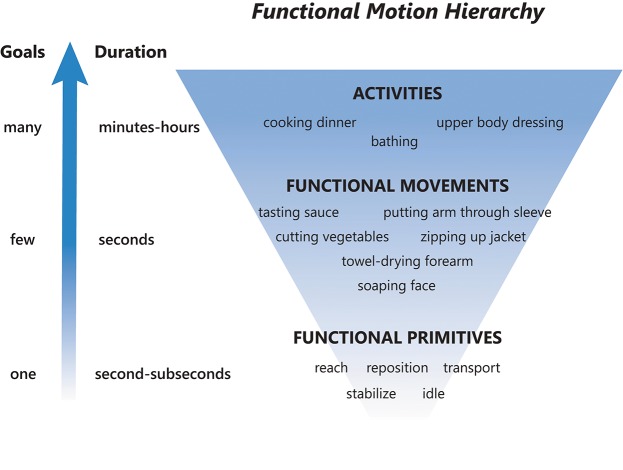
Functional motion hierarchy, adapted from Fanti (16). Functional motion can be broken down into levels of motion with decreasing durations and complexity. Activities are long-duration motions with many goals, functional movements are moderate-duration motions with a few goals, and functional primitives are short-duration motions or minimal-motions with one goal. A sequence of functional primitives combine to make a functional movement, and a sequence of functional movements combine to make an activity.

To provide a framework for discussion, we abstract functional motion in a hierarchical manner based on decreasing time scales and goals (Figure 1) (9, 10, 16) as follows:

- Activity: a sequence of motions that achieves several goals to accomplish one overarching purpose. These are complicated motion events on an extended time scale, occurring over minutes to hours (16). Examples: dressing, cooking dinner, bathing.
- Functional movement [also called actions; (16)]: a sequence of motions that achieves a few goals to accomplish a single purpose (7, 18). These are moderately complicated motion events on a medium time scale, occurring over seconds (16). A functional movement has been operationalized as a reach to grasp, action on, and release of an object (6). Examples: zipping up a jacket, tasting a spoonful of sauce, soaping one's face.
- Functional primitive [also called movemes; (8, 10, 16)]: a single motion or minimal-motion that achieves one goal. These are simple motion events on a short time scale, occurring over milliseconds to seconds (16). Primitives are elemental motions that cannot be further decomposed by a trained human observer. Examples: reaching, transporting, repositioning. We note that the term “primitive” has also been used to describe even more granular kinematic, kinetic, and neural elements of motion (19). Although these elements can combine to form functional primitives, they are not used for classification here as their identification requires instrumentation of the body [e.g., marker-based systems or computer vision to extract effector kinematics (20), EMG to extract muscle activation (21)] or instrumentation of the central nervous system [e.g., electrodes to extract neural activity (22, 23)]. We sought to develop a taxonomy that can be applied using human observation of real-time or videotaped motion.
- Importantly, activities and functional movements can be broken down into functional primitives and are targets for decomposition by the taxonomy.

### Taxonomy Structure

**Figure 2:**
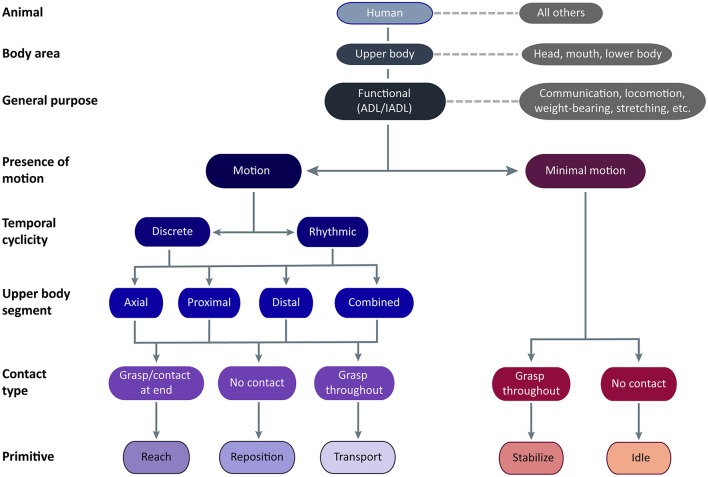
Functional UE motion taxonomy. The taxonomy is meant for application in humans using their upper body to perform functional activities. Classes of functional motion are characterized by the presence of motion, the temporal cyclicity of motion, the upper body segment primarily translating the UE, and the type of object contact. Importantly, grasp may occur anywhere on the UE, not just at the hand. Functional primitives are identified by their motion and contact type. The primitives are *reach* (motion to move into contact with a target object), *reposition* (motion to move near to a target object), *transport* (motion to convey a target object), *stabilize* (minimal-motion to keep a target object still), and *idle* (minimal-motion to stand at the ready).

The functional UE motion taxonomy is outlined in Figure 2. The taxonomy classifies the dominant motion phenotypes that are readily observable and explicit. In Linnaean fashion, we clustered motions into groups based on shared defining features and subdivided groups based on distinguishing features. We describe our logic regarding these features, and entities that the taxonomy does *not* cover, as follows:

#### Animal of Interest

We first classified motion in the animal of interest, focusing on humans. Although not the focus of the taxonomy, we expect that aspects of it may also apply to other animals capable of functional motion, despite differences in neural and peripheral effectors (24). For example, the reaching to and transport of food achieved by the forelimbs of a rat, the raptorial legs of a praying mantis, the claws of a crab, and the pseudo-articulation of octopus legs (9) share a similar phenotype and purpose as their human counterparts.

#### Body Area Performing Motion

We next classified the general region of the body performing the motion, focusing on motion that displaces the UE relative to the pelvis. Upper body motion may be actuated from within the limb with/without contributions from the trunk. Although motions executed by the head/eyes, mouth/tongue, and lower extremities may have similar purposes, they have distinct phenotypes and are not further considered here.

#### General Purpose of Motion

We next classified the general purpose of the motion, focusing on functional motions. These enable the engagement of objects or targets in the environment to support ADLs (25). Although these motions are termed “functional,” we readily acknowledge that other UE motions serve alternative functions. These “non-functional” motions include signing, gesturing, or making noise (e.g., tapping, snapping, clapping) to communicate concepts and emotion; arm swinging to increase ambulation efficiency (26); postures to bear the weight of the trunk or head; stretching; swimming, crawling, and gliding (i.e., wingsuit) to locomote; motions to alter the center of gravity to maintain balance; motions for reproduction, defense, and aggression; motions to convey artistic creation or narrative (e.g., dance, puppetry); and motions to play games or sports. While taxonomy definitions could overlap with some of these “non-functional” motions, the taxonomy was not designed to exhaustively account for all motion phenotypes elicited by their many purposes and targets.

#### Presence of Motion

We next classified the presence of motion, focusing on motions that translate the UE in space relative to the pelvis versus those with minimal motion. We include minimal motion in the taxonomy because of its ubiquitous presence in functional activities. Beyond object stabilization, we commonly observed the UE held stationary adjacent to the workspace in configurations suggesting isometric muscle contraction. For example, the shoulder could be elevated and abducted while the hand lay motionless on a surface, or the entire UE could be held aloft. This state especially occurs during transitions from bimanual to unimanual task execution (by the other UE). We exclude “rest” from the taxonomy because it does not directly contribute to functional engagement of an object, leaving it to describe true atonic motionlessness that is typically seen at the very beginning or very end of the task. For determination of minimal motion, we considered if UE location remained largely unchanged for ≥50 ms. We disregarded subtle drift in the UE held aloft, as long as there was no apparent target of the slight motion.

#### Temporal Cyclicity of Motion

We next classified the temporal cyclicity of the motion, focusing on motions that are discrete versus rhythmic. The temporal nature of motion is likely mediated by different neural substrates (27, 28). Discrete motions are monophasic, with a single motion from one object or location to another. Rhythmic motions have regular, repeated cycles of motion on a fixed target, with similar repeating amplitudes and directions. Within a bout, rhythmic motions are not observably different from one cycle to the next. Separate bouts of rhythmic motions are identified by a pause that exceeds between-cycle pauses, a change in UE configuration (often accompanied by a target change), or a change in motion amplitude or frequency. Rhythmic motions typically consist of *reach*-*transport* cycles (e.g., unscrewing a bottle cap, entailing the repeated reach to grasp the cap and transport to turn it) and *transport*-*transport* cycles (e.g., tooth brushing, entailing repeated transports of the toothbrush across the teeth in one direction and the opposite direction).

#### Upper Body Segment Performing Motion

We next classified where upper body motion is predominantly occurring, focusing on the upper body segment that is primarily acting to translate the UE in space. Motion at three upper body segments is considered: distal (forearm, wrist, and/or hand), proximal (shoulder and/or elbow), or axial (trunk). Location of motion is defined by the effector, not its actuator; for instance, we consider shoulder elevation a proximal motion even though it is actuated by muscles on the trunk. Typically, one segment performs the majority of UE translation, but occasionally adjacent segments act together to contribute to UE translation. For example, reaching beyond arm's length requires both axial and proximal segments for trunk flexion, shoulder flexion, and elbow extension. Scraping the inside of a jar requires both the proximal and distal segments for shoulder adduction and forearm supination. We disregard passive segmental motion brought about by motion at another segment, for example, translation of the hand in space by the proximal motion of shoulder flexion and elbow extension. During proximal motions, we also subsume distal motions that orient or shape the hand in preparation for object contact, as these are not the dominant source of UE translation.

#### Contact Type

We next classified motion by the type of object contact, identified by the touch of at least one surface of the end-effector (12). Contact may result in a prehensile grasp, which uses at least two contact points (typically fingers) generating opposing forces to secure the object to the UE (12, 13). Contact may also result in a non-prehensile grasp, which uses one generated force and an opposing external force (e.g., from gravity, a stable surface, or the opposite UE) to secure the object. For example, supporting a tray is a non-prehensile grasp because the UE generates upward force and gravity generates an opposing downward force to secure the tray. Importantly, we are not defining grasp in a traditional, hand-centric sense; grasp may occur anywhere on the UE. For example, a shopping bag held in the crook of the arm would be considered a non-prehensile grasp. Entities that envelop the UE, such as clothing or fluids, would similarly be held in a non-prehensile grasp if they are the target of engagement. Of note, the UE may contact an object in the workspace (e.g., table surface, piece of paper, keyboard) without it being a target of engagement. The observer must disambiguate whether the UE is positioned for a non-prehensile grasp (e.g., applying force to keep a magazine from flipping shut) or is contacting the object without targeted action (e.g., touching a keyboard lightly between bouts of typing). We make the distinction by inferring whether the object would move (i.e., the magazine page) if contact were removed.

#### Functional Primitives

Incorporating these motion features, we classified motion into five functional primitives, based on their phenotype and purpose. Major distinguishing phenotypic features are presence of motion and type of object contact, while temporal cyclicity and segmental motion serve to cluster primitives into families. We delineate their beginnings and endings for data segmentation purposes (Table 1), and demonstrate their appearance in labeled videos (Supplementary Materials). Primitives are defined as follows:

- *Reach*: the purpose is to make contact with a target object. Motion is present. Object contact at the end may result in grasp or touch.
- *Reposition*: the purpose is to move into proximity of a target object. Motion is present. There is no object contact. The UE may be moving away from the target object or toward it from another location. Motion away from a target object and toward the body, sometimes termed “retraction,” would be considered a subtype of *reposition*.
- *Transport*: the purpose is to convey a target object in space. Motion is present. There is grasp or touch of a target object throughout the motion. We include motions that engage objects that appear largely stationary, such as scratching a limb, sweeping or smoothing a surface, or swiping a device screen. In these cases, elements on the object surface (e.g., epidermis, dust, virtual interface) are the target objects conveyed in space.
- *Stabilize*: the purpose is to hold a target object still, such that removal of UE would result in object movement. Motion is minimal. There is grasp of a target object throughout the minimal-motion.
- *Idle*: the purpose is to stand at the ready near a target object, often out of the way of motion of the opposite UE. Motion is minimal. To us, the apparent readiness of the UE to join the activity was reminiscent of a car idling at a stoplight—turned on and prepared to engage, but motionless. The UE is located on a surface, at one's side, or held aloft in a stationary configuration. There is no grasp of the target object, although contact of non-target objects may be present. In these instances, *idle* is distinguished from the similarly appearing *stabilize* by the low likelihood of object movement should the UE be removed.

**Table 1:** Functional Primitives and their characteristics.

**Primitive**	**Purpose**	**Presence of motion**	**Type of object contact**	**Primitive start**	**Primitive end**
Reach	To move into contact with a target object	Motion present	Grasp or touch at end^**^	When UE starts moving	When object contact completed (if grasp, all fingers in full contact)
Reposition	To move proximate to a target object	Motion present	No grasp or contact	When UE starts moving	When UE becomes stationary
Transport	To convey a target object	Motion present	Grasp throughout	When UE starts moving	When grasp released or new direction of motion begins with target object
Stabilize	To keep a target object still	Minimal motion^*^	Grasp throughout	When UE stops moving	When grasp released or motion begins with target object
Idle	To stand at the ready near target object	Minimal motion^*^	No grasp or contact	When UE stops moving	When UE starts moving or when target object is inserted into hand

Functional primitives are identified by the presence of motion and type of object contact. Motion is the translation of UE in space relative to a body coordinate frame centered at the pelvis. The type of object contact could be a prehensile or non-prehensile grasp or non-grasp contact. We operationalize primitive beginnings and endings for data segmentation purposes.

* Minimal motion may entail slight UE configuration drift that has no apparent target.

** A non-target object may be held in the hand during a reach to a new target object.

We also classify the failed attempt to accomplish some primitives, which we term an “incomplete” variant. These are identified less by their motion phenotype than by an observer inferring what an individual was trying to do. Cues include a target object falling from the hand (incomplete *transport* or *stabilization*) or the UE moving directly toward a target object but not contacting it in one motion (incomplete *reach*). Subsequent primitives are typically *idles*. We distinguish an incomplete *reach* from a normal *reposition* by their apparent trajectories: an incomplete *reach* will typically aim toward a target object, whereas a reposition typically ends vaguely nearby. Other cues include some degree of hand pre-shaping (if the individual is capable) and orientation of the eyes/head toward the target object during an incomplete *reach*. We cannot distinguish a normal from an incomplete *reposition* or *idle*. Of note, though the “incomplete” variant indicates a failed motion/minimal-motion contributing to activity limitation, it does not read out the severity, location, or modality of impairment.

We also note a few modifications to our definitions. The first is when the target object envelops the UE (i.e., water, blown air, clothing). When the target object is enveloping, the UE may extend through it following initial contact, for example when reaching through a stream of water or a shirt sleeve. In these instances, the end of *reach* is defined not by initial contact but by when UE motion ceases or there is a directional change. In addition, the UE may make motions/minimal-motions to engage the enveloping object (e.g., rinsing in water) or to orient the object to facilitate engagement by the other UE (e.g., to adjust clothing). In these cases, the enveloped UE is *stabilizing* or *transporting* the object with a non-prehensile grasp (see dressing video, Supplementary Materials).

The second modification is when one of the UEs is itself be the target object to be acted on by the other UE, for example to be scratched, soaped, lotioned, or toweled off. Motions/minimal-motions made by the acted-on UE facilitate access for the acting UE. These are conceptually similar to motions/minimal-motions made when an object is held by one UE to be acted on by the other. The target object is thus the focal area of epidermis that is *transported* or *stabilized*. There is no grasp, however, as the epidermis is integral to the UE.

The third modification is when the UE is dual-tasking, for example when the hand grasps an object while engaging another. For example, a pen may be reoriented out of the way in the hand while the UE retrieves and opens a book. The focus of functional engagement is the book, not pen; thus the primitives are *reach, transport*, and *stabilize* related to the book, not a series of *transports* related to the pen. It is our sense that the held object is on standby, kept in the hand to expedite later re-engagement. The observer must thus determine whether an object in hand is the primary focus of the ongoing motion. This extends to objects worn on the body, such as a wristwatch or clothing; after donning, they are seldom the focus of engagement. If, however, one extends the attired UE toward the other UE so that clothing can be adjusted or the wristwatch removed, this motion would be considered a *transport*. This is conceptually akin to *transporting* the UE to the other limb so that it can be acted on, in the cases of lotioning, scratching, etc. noted above.

### Assessing Taxonomy Validity

We developed the taxonomy using videotaped recordings of 16 subjects (healthy and stroke-impaired) performing various ADL-like tasks; an early iteration has been previously described (29). For the refined taxonomy, we examined validity and reliability using new motion data generated by healthy and stroke-impaired subjects (see Table 2 for demographic and clinical details) performing various activities (see Table 3 for task parameters). Written informed consent was obtained from participants. Two video cameras (Ninox 125) were positioned ~1 m orthogonal to the activity. Subjects also wore nine inertial measurement units (IMUs; Noraxon) affixed to their pelvis, thoracic (T10) and cervical (C7) spine, and bilateral arms, forearms, and hands. Video and IMU data were synchronously recorded at 100 Hz.

**Table 2:** Subject demographics for validity and reliability assessments.

**Subject type**	***N***	**Gender (M:F)**	**Age (years)**	**FMA score**	**mRS score**	**Time since stroke (years)**
**A. TAXONOMY VALIDATION AND APPLICATION**						
Stroke	9	4:5	50.3 (42.6–70.2)	49.6 (26–61)	2.4 (2–4)	11.4 (0.5–38.4)
**B. MACHINE LEARNING VALIDATION**						
Healthy control	5	2:3	28.1 (19.0–43.3)	N/P	N/P	N/A
**C. TAXONOMY RELIABILITY ASSESSMENT**						
Stroke	7	3:4	51.4 (42.6–70.2)	47.1 (26–61)	2.5 (2–4)	7.7 (0.5–11.7)

*All subjects were right-handed (premorbidly for stroke patients), as determined by the Edinburgh Handedness Inventory. All stroke patients were right-side paretic. Age, FMA score, and time since stroke are average with range in parentheses. The FMA measures UE motor impairment, with maximum score of 66 indicating fully normal movement. The mRS measures disability, with a score of 0 indicating no symptoms and 6 indicating death. Labeled data from seven of the nine stroke subjects in **(A)** were used for **(C)**. FMA, Fugl-Meyer Assessment; mRS, modified Rankin Scale; N/P, not performed; N/A, not applicable*.

**Table 3:** Activity battery for validity, reliability, and application assessments.

**Activity**	**Trials**	**Workspace**	**Target object(s)**	**Instructions**
**A. INTERRATER RELIABILITY ASSESSMENT AND APPLICATION**				
Tooth brushing	5	Sink with toothpaste and toothbrush on either side of the countertop, 30 cm from edge closest to subject	Travel-sized toothpaste, toothbrush with built-up foam grip, faucet handle	Apply toothpaste to toothbrush, wet with water, brush teeth, rinse toothbrush, place toothbrush back on countertop, replace cap on toothpaste tube
Washing face	5	Sink with a small tub in it and two-folded washcloths on either side of the countertop, 30 cm from edge closest to subject	Washcloths, faucet handle	Fill tub with water, dip right-side washcloth into water, wring it, wash each side of face, place washcloth back on countertop Use left-side washcloth to dry face, place washcloth back on countertop
Hair combing	5	Tabletop with comb placed at midline, 25 cm from edge closest to subject	Comb	Pick up comb and comb both sides of head
Applying deodorant	5	Tabletop with deodorant placed at midline, 25 cm from edge closest to subject	Deodorant	Remove cap, twist base, apply deodorant to each armpit, replace cap, and place deodorant on table
Don/doffing glasses	5	Tabletop with glasses placed at midline, 25 cm from edge closest to subject	Glasses	Put on glasses, return hands to table, remove glasses and place on table
Drinking	5	Tabletop with water bottle and paper cup 18 cm to the left and right of midline, 25 cm from edge closest to subject	Water bottle (12 oz), paper cup	Open bottle, pour water into cup, replace cap on bottle, take a drink, place cup on table
Eating	5	Table top with a standard-size paper plate (at midline, 2 cm from edge), utensils (3 cm from edge, 5 cm from either side of plate), a baggie with a slice of bread (25 cm from edge, 23 cm left of midline), and a margarine packet (32 cm from edge, 17 cm right of midline)	Fork, knife, re-sealable sandwich bag, slice of bread, single-serve margarine container	Remove bread from sandwich bag and bring to plate, open margarine contained and spread on bread, cut bread into quarters, cut off and eat a bite-sized piece
**B. MACHINE LEARNING VALIDATION**				
Reach/transport (HV)	3	Horizontal circular array (48.5 cm diameter) of 8 targets (5 cm diameter)	Toilet paper roll	Reach: reach between 2 rolls placed 180° across Transport: move roll between targets 180° across

*Activity parameters include the workspace set-up, objects of focus, and task instructions. Subjects were positioned relative to the workspace so that they could reach the furthest target object at full UE extension (non-paretic side if stroke). This ensured that the workspace would not normally provoke compensatory movement. All subjects were instructed to use their right UE (the paretic side for stroke)*.

The most important validation step was confirming the taxonomy could account for all functional UE motion occurring during functional activities. Nine chronic stroke patients with right-sided hemiparesis (Table 2A) performed seven ADL tasks: tooth brushing, face washing, hair combing, applying deodorant, donning/doffing glasses, drinking, and feeding (Table 3A). Trained coders focused on only right-sided motions and segmented the resulting 160 min of video data. We confirmed that all functional motions could be classified using the taxonomy, although we note that our testing setup (humans performing UE-based ADLs) predetermined the classification of animal, body area, and general purpose. We did not encounter motion that required a “non-classifiable” option available to coders. However, we note that recordings began after subjects stopped conversing with investigators, when communication gestures (non-classifiable by our taxonomy) were typically prevalent. In real-world settings, it is expected that there will be gaps in the application of the taxonomy when motions for other purposes, such as communication or weight-bearing (Figure 2), are distributed among functional motions.

We next confirmed a subset of our classification intuitions using machine learning and a challenging discrimination task. Taxonomy construction is inherently subjective: humans select the major distinguishing features of each cluster, and purposefully ignore inter-individual differences (such as gender, movement speed, or arm length) that do not inform the classification of interest. To begin to probe the plausibility of our intuitions, we examined if the motion phenotypes of two primitives—*transport* and *reach*—are objectively different, as viewed by an unbiased machine learning algorithm. We chose these two primitives because the similarity of their motions should be maximally challenging to discriminate, thus providing a conservative appraisal of our decision-making. We first evaluated whether the algorithm could distinguish between the primitives; a high classification performance would indicate that their phenotypes are different enough that it would be reasonable to call them by different names. Second, we examined the motion characteristics upon which the algorithm was basing its decisions. In separating *transport* from *reach* primitives, we humans intuit that an important phenotypic distinction is the presence of object grasp throughout the motion (*transport*) vs. just at the end (*reach*). If similar information is used by the algorithm for its identification, this would indicate that our intuition is acceptable.

We had five healthy controls perform proximal *reaches* and *transports* in a task that controlled for target locations and heights (Tables 2B, 3B). We used this task and healthy controls, as opposed to the functional activities and stroke patients, because we wished to generate nearly identical UE motions. The similarity of these motions thus challenged the algorithm to identify subtle phenotypic differences between the primitives, which may otherwise be exaggerated in the less controlled setting of functional activities. Proprietary software (myoMOTION, Noraxon USA) generated joint angles from the recorded IMU data.

Trained coders used the video data to label the *transport* and *reach* primitives, which simultaneously labeled the joint angle data. Whole primitives were used to train a binary decision tree (30). CSV files containing their joint angle data and their labels were imported using a custom Matlab code, converted to a numeric matrix, and provided to the binary decision tree. Besides the joint angles and labels, no additional information was given to the algorithm. The tree was trained with 60% of the data and tested with the remaining 40%, repeated 100 times, and the resulting classification accuracies were averaged. The algorithm discriminated *reaches* from *transports* with 92.1% accuracy. Inspecting the nodes of the binary tree, we found that the algorithm identified *reaches* by their greater wrist extension, wrist supination, and elbow extension. *Transports* had less wrist extension/supination and more shoulder flexion and abduction. These informative features correspond to motion phenotypes that orient and position the hand to engage in eventual grasp (*reach*) or that keep the distal segment stable while clearing the table with a grasped object (*transport*).

### Assessing Taxonomy Reliability

Finally, we used Cohen's kappa (31) to determine the inter-rater reliability of classification with this taxonomy. Three trained coders and an expert (AP) independently segmented videotaped motion data from seven of the stroke subjects performing the activities noted above (Tables 2C, 3A; the remaining two stroke subjects were coded by the expert alone and were therefore unavailable for analysis). Between the coders and expert, we compared the labels of the primitives and the time points of their transitions (on/offsets). We examined a window of ±20 ms around the transitions identified by the expert. Coder labels were considered incorrect if: (1) transition points mismatched by greater than ±20 ms, even if primitive labels matched; (2) transition points were within the ±20 ms window, but primitive labels mismatched; or (3) if both transition points and labels mismatched. Kappa values were averaged across coder-expert pairs for each primitive. Agreement was excellent for primitive labels, indicated by high kappa values: *reach*, 0.97; *reposition*, 0.96; *transport*, 0.96; *stabilize*, 0.95*; idle*, 0.99. The main, albeit uncommon, source of error came from correctly labeled primitives that mismatched the transition points of the expert.

### Application of the Taxonomy

We next demonstrate the use of the taxonomy to decompose rehabilitation-like activities into their constituent primitives and overarching motion characteristics. We evaluated seven ADL tasks (Table 3A) performed by nine stroke subjects (Table 2A).

**Figure 3:**
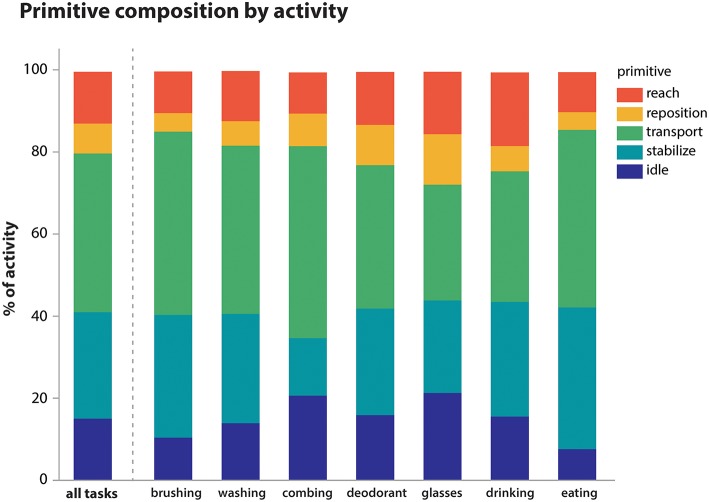
Primitive composition of activities. Primitives were summed within activity irrespective of temporal cyclicity or upper body segment. Their average proportional contribution to each activity is shown. Overall, *transports* (38.7%) and *stabilizations* (26.0%) predominated across activities, while *reaches* (12.5%), *idles* (15.0%), and *repositions* (7.3%) were less prevalent.

Using the videotaped data, coders segmented the activities into primitives, noting also the presence of motion, its temporal cyclicity, and the upper body segment effecting the motion. A total of 11,223 primitives was identified. We examined the overall primitive dose (total repetition count), primitive density (repetitions/time), and the percentage of primitive type per activity (Table 4; Figure 3). As expected, longer-lasting activities had higher primitive doses, with feeding having the highest average dose of 81.4 primitives. Primitive density was fairly consistent across activities, about 1 primitive/second (Table 4). Primitive type varied in the sampled activities (Figure 3), but *transports* and *stabilizations* predominated across activities (38.7 and 26.0% on average, respectively), and *reaches* and *idles* were less prevalent (12.5 and 15.0%, respectively). *Repositions* were the least prevalent (7.3%). Finally, we observed that the “incomplete” variant was fairly infrequent, accounting for 0.5% of primitives on average (not shown).

**Table 4:** Primitive decomposition of ADL-like activities.

**Activity**	**Primitive dose (count)**	**Duration (s)**	**Primitive density (primitive/s)**
Brushing teeth	43.6 (16.6)	67.1 (25.2)	0.7 (0.3)
Washing face	38.3 (14.5)	41.5 (16.1)	1.0 (0.4)
Combing hair	14.0 (5.1)	15.6 (6.2)	0.9 (0.3)
Applying deodorant	26.8 (8.7)	24.7 (8.6)	1.2 (0.4)
Don/doffing glasses	21.8 (7.0)	22.1 (8.0)	1.0 (0.3)
Drinking	24.8 (11.4)	33.2 (14.4)	0.8 (0.3)
Feeding	81.4 (34.6)	88.4 (45.1)	1.1 (0.5)
All	35.8 (14.0)	41.8 (17.6)	1.0 (0.3)

*Nine individuals with chronic stroke performed the tasks. Primitive dose and task duration were obtained from labeling the videotaped data. Average values with SD in parentheses are shown*.

**Figure 4:**
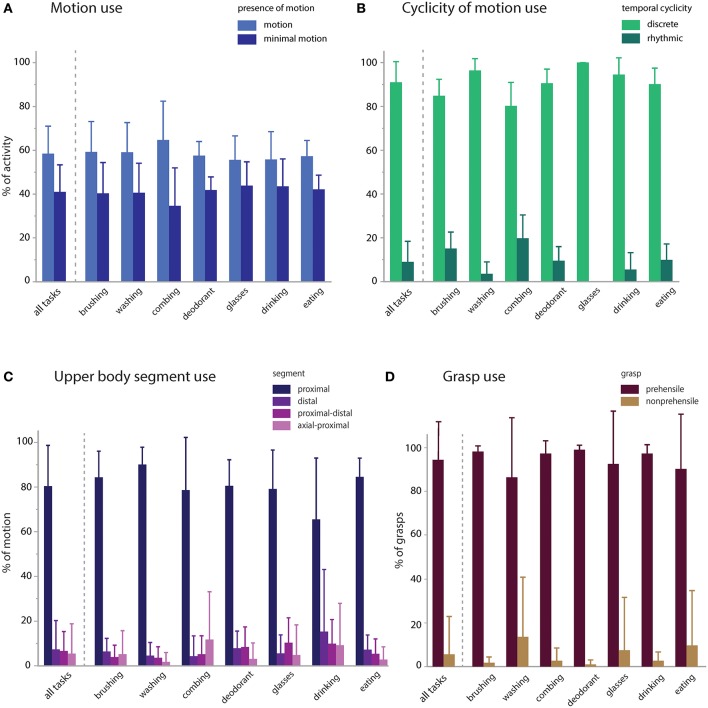
Motion characteristics of activities. Primitive features were used to characterize the proportional contribution of motion, temporal cyclicity, and upper body segment to the execution of the activities. **(A)** Presence of motion: activities were executed with modestly more functional motion (58.5%) than minimal-motion (41.0%). **(B)** Temporal cyclicity of functional motion: activities were primarily executed with discrete motions (91.0%) and less commonly with rhythmic motions (9.0%). **(C)** Upper body segment effecting functional motion: activities were primarily executed by the proximal segment (80.4%), and less by the distal (7.4%), proximal-distal (6.7%), axial-proximal (5.5%), or axial (0%) segments. **(D)** Grasp use in *transports* and *stabilizations*: activities were primarily executed with prehensile grasps (94.5%) and uncommonly with non-prehensile grasps (5.5%).

We then calculated the proportional contribution of motion characteristics to activity execution (Figure 4). Activities were mostly comprised of functional motion (58.5% of the sampled data, on average) but also had a substantial contribution of functional minimal-motion (41.0% on average; Figure 4A). Within the functional motion category, discrete motions predominated (91.0% on average) with rhythmic motions occurring infrequently (9.0% on average; Figure 4B). Rhythmic motions were more pronounced in specific activities such as tooth brushing and hair combing (15.1 and 19.7% of each activity, respectively). UE translation was predominantly effected by the proximal segment (80.4% on average), with minimal contribution of the distal segment or combined segments (<8% each, on average) and no observations of axial-only functional motions (Figure 4C). For primitives engaged in object grasp (*transport, stabilize*), prehensile grasp was used most commonly (94.5% of contacts, on average), with minimal use of non-prehensile grasp (5.5% on average; Figure 4D).

**Figure 5:**
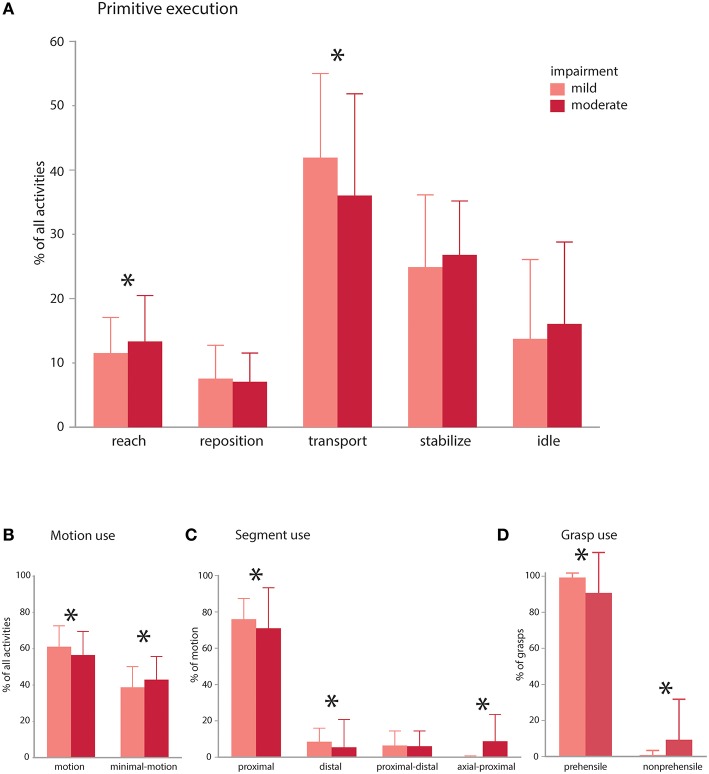
Impairment effects on primitive composition and motion characteristics. Patients with mild (average FMA 59.5) and moderate (average FMA 41.6) impairment were compared. **(A)** Primitive execution: Mildly impaired subjects used more *transports*, whereas moderately impaired patients used more *reaches*. There was a trend for more *idles* and *stabilizes* in the moderately impaired group. **(B)** Motion use: mildly impaired patients used more functional motion to execute the activities, whereas moderately impaired patients used more minimal-motion. **(C)** Segmental use: mildly impaired patients more often used their proximal and distal segments to execute the activities, whereas moderately impaired patients more often used their axial and proximal segments in combination. **(D)** Grasp use: mildly impaired patients more often used prehensile grasps whereas moderately impaired patients more often used non-prehensile grasps. Mildly and moderately impaired patients had comparable use of discrete and rhythmic motion (not shown). *p < 0.05.

In the same subjects, we evaluated whether impairment impacts the primitive composition or motion characteristics of the activities (Figure 5). We compared mildly vs. moderately impaired subjects [average Fugl-Myer assessment (FMA) scores 59.5 (*n* = 4) and 41.6 (*n* = 5), respectively; (32)]. For the comparisons, we used a general linear mixed model regression to analyze classified data from the right-paretic UEs, with impairment category as a fixed effect and subject as a random effect.

The impairment groups achieved comparable doses of primitives across activities (mild: 36.3 vs. moderate: 35.4; NS), but mildly impaired patients achieved a higher primitive density [mild: 1.2 primitives/s vs. moderate: 0.9 primitives/s; *t*_(308)_ = 9.46, *p* < 0.0001]. In the activities, mildly impaired patients used more *transports* [mild: 41.9% vs. moderate: 36.0%; *t*_(308)_ = 2.54, *p* < 0.001], whereas moderately impaired patients used more *reaches* [mild: 11.5% vs. moderate: 13.3%; *t*_(308)_ = 2.45, *p* = 0.015; Figure 5A]. Moderately impaired patients also showed a trend for more *idles* [mild: 13.7% vs. moderate: 16.1%; *t*_(308)_ = 1.64, *p* = 0.10) and *stabilizes* [mild: 24.9% vs. moderate: 26.8%; *t*_(315)_ = 1.7, *p* = 0.089]. *Repositions* were comparable across groups (mild: 7.5% vs. moderate: 7.1%; NS). Though uncommon, there was a trend for more incomplete primitives in the moderately impaired group [moderate: 0.68% vs. mild: 0.32%; *t*_(308)_ = 1.96, *p* = 0.051].

Evaluating the motion characteristics of activity performance, we found that mildly impaired patients used more functional motion to complete the activities [Figure 5B; mild: 61.0% vs. moderate: 56.4% of the data, *t*_(315)_ = 3.27, *p* = 0.0012], whereas moderately impaired patients used more functional minimal-motion [moderate: 42.8% vs. mild: 38.6%; *t*_(315)_ = 3.04, *p* = 0.003]. Examining the characteristics of functional motion, we found that temporal cyclicity was comparable across groups, not only for discrete motions (mild: 89.1% vs. moderate: 88.5%; NS) but also for rhythmic motions (mild: 10.8% vs. moderate: 11.5%; NS). We also evaluated how patients achieved UE translation, finding that mildly impaired patients more often used their proximal segment [Figure 5C; mild: 83.7% vs. moderate: 77.9%; *t*_(308)_ = 2.82, *p* = 0.005] and distal segment [mild: 9.3% vs. moderate: 5.8%; *t*_(308)_ = 2.44, *p* = 0.015]. Conversely, moderately impaired patients more often used axial-proximal motion [moderate: 9.8% vs. mild: 0.1%; *t*_(308)_ = 6.84, *p* < 0.0001]. Both mild and moderate impairment groups used proximal-distal motions comparably (mild: 6.9% vs. moderate: 6.5%; NS). In terms of grasp types used for *transports* and *stabilizations*, mildly impaired patients used more prehensile grasps [Figure 5D; mild: 99.2% vs. moderate: 90.6% of all grasps, *t*_(309)_ = 4.88, *p* < 0.0001] whereas moderately impaired patients used more non-prehensile grasps [moderate: 9.3% vs. mild: 0.8%, *t*_(309)_ = 4.88, *p* < 0.0001].

## Discussion

To date, the mechanistic and therapeutic study of functional motion has been limited by the absence of an organizing framework with a common nomenclature. Here, we generated a taxonomy for classifying upper extremity functional motion in humans. The taxonomy focuses on object-related motions and minimal-motions that contribute to daily function. The taxonomy represents a hierarchical classification approach that organizes classes of motion within larger classes. Based on phenotype, we provide an inventory of functional primitives with objective, mutually exclusive characteristics. The resulting systematic nomenclature is important for universal communication, contextualization of findings, and clinical applications.

We validated the taxonomy by first confirming that it was capable of comprehensive motion classification. In a selection of typical rehabilitation activities, the taxonomy could parse and account for all observed UE motion performed by stroke patients.

Because taxonomies are subjective, in that a human decides the dominant features that unite or divide groups, we sought to confirm our intuitions with an objective classification approach. With supervised machine learning, we showed that *reaches* could be objectively distinguished from *transports* despite their phenotypic similarity. With feature selection analysis, we found the algorithm based its distinction on kinematic features related to grasp preparation (*reach*) or to stable conveyance of the object (*transport*). Although no grasp data were available because the fingers were not instrumented with IMUs, the algorithm based its decision on UE configurations related to anticipated or ongoing object grasp, similar to us. This suggests that our taxonomic approach, at least for distinguishing *reaches* from *transports*, extends beyond face validity. We note that inspection of all classification branch points in the taxonomy was not done and requires additional investigation.

We also showed that inter-rater reliability for primitive classification was very high among trained coders, with reliable notation of primitive beginnings and endings. This indicates that the taxonomy is intuitive and can be readily used to classify and segment continuous functional motion data.

### Application of the Taxonomy

We demonstrated how the taxonomy can be used for the quantitative appraisal of rehabilitation activities, first by decomposing them into primitives. As expected, primitive dose scaled with activity duration, though primitive density remained fairly constant, underscoring the regularity of movement speed across individuals (33). Primitives that directly engage an object (*transports, stabilizations*) were the mainstay of the activities, while primitives that move into contact with, move near to, or are stationed nearby an object (*reaches, repositions, idles*) had supporting roles. Knowing primitive content of an activity is an important step toward unpacking the “black box” of rehabilitation (4). Quantifying primitives allows investigators to identify precisely *what* and *how much* was trained, regardless of which activities generated them. This is not only critical for informing a dose-response relationship, but is also vital for reproducibility and standardized implementation of rehabilitation interventions. From a clinical standpoint, information about the estimated dose and proportion of primitives in functional activities may help guide activity selection for training.

Second, by examining the motion characteristics of the activities, we found that paretic UE functionality did not mean nonstop motion; purposeful minimal-motion also played a substantial role. This observation implies that approaches incrementing motion alone, as with wrist-worn accelerometers, may underestimate functional UE use. Furthermore, we found that proximal motions greatly outnumbered distal motions. It is possible that distal motions were undersampled due to paresis, but we noted no distal impairment bias in our sample upon inspection of FMA subscores. Prehensile grasps also greatly outnumbered non-prehensile grasps in the activities. These findings underline the importance of training both the arm and hand after stroke, as both contribute to UE function (34): proximal motion precisely translates the hand in space, which then dexterously engages objects.

Finally, by assessing the primitives and motion characteristics of patients with different levels of impairment, we found differential functional strategies despite the achievement of comparable primitive doses. Moderately impaired patients used their right paretic UE for more *reaches*, which we observed as repeated distal reaching and re-grasping of an object to secure it. Mildly impaired patients used more *transports*, whereas moderately impaired patients showed a trend for more *idles* and *stabilizations*. This result may reflect dominance-switching that can occur with dominant limb impairment (35). We note that although some differences were significant, they were also small, possibly because the moderately impaired group was at the milder end of its impairment range (32). We also note that the taxonomy does not read out the kinematic quality of the performed primitives, which might be expected to reveal larger group-wise differences (36).

Moderately impaired patients also used less proximal and distal motion and more axial-proximal motion to execute the activities, indicating increased compensatory behavior from reduced limb excursion. Similarly, moderately impaired patients used more non-prehensile grasps, indicating the adoption of alternative strategies (such as pinning an object between UE and table) to secure it. Finally, moderately impaired patients showed a trend for more incomplete primitives, although they were uncommon in both groups. We speculate different reasons for each group: those with mild impairment were able to successfully perform *reach* and *transport* primitives, whereas those with moderate impairment switched dominance roles (37) to enable the successful performance of *idles* and *stabilizations*. While these characterizations may seem clinically intuitive, they demonstrate the taxonomy's ability to quantitate subtle differences in functional strategy. Importantly, these characterizations are limited to the activities that were examined, and do not generalize to all ADLs. For example, others have noted more non-prehensile grasps in general, but sampled a wider array of activities (38).

### Previous Work in Motion Classification

Current approaches for characterizing human motion tend to be either granular or broad. At the lowest level, kinematics are used to describe UE segmental positions and joint angles and their time derivatives. Although primitives such as a *reach, transport*, or *stabilization* are commonly used to generate kinematics, motion classification has not historically been its goal. We view the characterization of primitives and kinematics as complementary. Kinematics are analogous to the tissue structure of a species, generating the phenotype that helps define the primitive (with underlying patterns of neural firing analogous to genotype).

Others have focused on an important component of primitives, the hand grasp and its actions (12, 13). These taxonomies comprehensively detail hand function, but neglect the remainder of UE motion and minimal-motion that contributes to function. Investigators are encouraged to pair these taxonomies with ours, as needed, to capture greater level of detail at the hand. An addended grasp taxonomy would enable the identification of the UE doing double-duty, for instance when an object is held in the hand for later use while a *reach, transport*, or *stabilize* is directed toward a separate object.

On the other end of spectrum, motion has been classified at the level of functional movement. A functional movement is a purposeful movement that accomplishes a functional goal (7, 18), defined as the reaching to, grasping, moving or manipulation, and releasing of an object (6). This definition does not account for *stabilizations* and *idles*, potentially underestimating functional UE use. In addition, functional movements may deviate from this definition, such as when they lack interleaved object releases and reaches. For example, tooth-brushing entails sequential *transports* of the toothbrush to the toothpaste tube, to the faucet, to the mouth, and within the mouth. In these cases, parsing motion into functional movements would be challenging.

Others have classified human motion at the level of activities, such as feeding oneself (39). Even if activities are identically named, their constituent motions can have variable type, number, and order, depending on target objects and goals. For example, the activity of cooking breakfast entails different motions if eggs or pancakes are made, a whisk or an egg-beater is used, or if eggs are beaten before or after the pan is retrieved. Along these lines, activity classification is confounded by cultural differences, where motions achieving the same activity are quite different, e.g., the making of a traditional Japanese versus Swedish breakfast. Classification at the level of activity thus may not be valid unless calibrated for different cultures (40, 41). To account for all of these variants of motion within the activity, an activity-based classification system would rapidly become congested and impractical. Tools such as the Assessment of Motor and Process Skills have alternatively focused on measuring the quality and sequencing of motions within activities (41). While this approach appraises performance of activity-specific subtasks, it does not offer a nomenclature that accounts for all constituent motions. It is important to have a system by which motions—regardless their activity or cultural context—can be parsimoniously but universally identified.

Functional primitives provide a means to decompose these functional movements and activities, but their orderly cataloging has not heretofore been undertaken. Most similar is Labanotation, developed to record UE postures and gestures for dance choreography (15). However, its application is not functionally relevant, and its graphical notation does not provide a semantic nomenclature. Others have classified *reaches* and “drawing” (effectively *transports*) using machine learning, but did not develop additional nomenclature (8). Although many of the terms in our taxonomy are used in everyday language, we are the first to systematically define them and organize them into a functional framework.

Beyond use in clinical applications, functional primitives may offer insights into the neural control of movement. In humans and other vertebrates, prolonged electrical stimulation of the brain and spinal cord elicits stereotyped and ethologically relevant motions. For example, motor and premotor cortical stimulation generates UE motions consistent with *reach, transport*, and *reposition* (42–44). These findings suggest that primitives may be neurally hard-wired and could be a central organizing principle of functional motion (43–45).

### Limitations

The taxonomy has several limitations to consider. First, it does not exhaustively describe the phenotype of functional motions; one cannot reverse engineer the kinematics of a primitive based on our descriptions. Thus, our definitions would be of limited use for roboticists and engineers seeking to generate anthropomorphic motion *de novo*. However, the role of a taxonomy is not to catalog all of an entity's characteristics in order to recreate it. For example, knowing the features that classify an elephant—an embryological notochord, vertebrae, mammary glands, and trunk—do little to describe its appearance. Rather, a taxonomy provides a logical scaffold to make sense of observed features, generating classes based on fundamental similarities and differences.

Along these lines, the taxonomy does not grade the abnormality of motion, nor should it necessarily do so. Biological taxa do not report on the appearance of its members beyond their shared fundamental phenotype—a hairless tiger, a three-legged tiger, and a healthy tiger are all still classified as *Panthera tigris*. Similarly, the taxonomy classifies motions with shared fundamental characteristics, regardless of whether they are jerky, use abnormal inter-joint coordination, are slow or inaccurate, or are sequenced incorrectly. Detailing the abnormality of a classified motion requires adjunctive means of motion assessment, such as kinematics (46, 47) or error-type analysis (41, 48). The role of the taxonomy is to identify and name the motion, whereas these complementary methods serve to qualify it.

Second, the taxonomy does not classify the extent of motion in peripersonal space, which may be neurally encoded (49). We suspect that motion extent may be captured in part by the upper body segment driving translation, with distal motions usually having smaller displacements and proximal motions usually having larger. It may also be important to identify motions ending in peripersonal space near to or far from the body, or above or below the body (49). The spatial characteristics of these classes and the boundaries between them require cluster analyses of positional data, which is ongoing in our laboratory.

Third, the taxonomy partially relies on an observer who understands the activity context and the role of each limb with respect to the behavioral goal. Based on experience, an observer has an internal model of the motions and target objects required to complete the activity. Classifying an “incomplete” variant is thus more informed by the failure to match this model than by its phenotype. Similarly, distinguishing an UE *idle* from a clothing *stabilization* requires knowledge about what the other UE is doing. The need for context is not uncommon in the classification of behavior. For example, the ethological meaning of teeth-baring in humans—threat, grimace, or smile—is informed by the social situation and recipient (50). For an observer without this knowledge, such as a machine learning algorithm, classification could be challenging.

Fourth, the taxonomy was developed in a limited set of activities, all of which required open kinematic chain motions with a stable center of gravity (25). With observation of a wider array of ADLs, we may find that weight-bearing postures and balance-maintaining motions need to be incorporated into the functional taxonomy. In the future, we anticipate that primitives will require additional sub-classification, creating a taxonomy with increased breadth and depth.

Finally, we confirmed that the taxonomy could account for all observed motions in our functional activity battery, which is the most important validation step. As a secondary step, we used an unbiased machine learning algorithm to confirm the taxonomy's phenotypic distinction between *reaches* and *transports*. While the algorithm supported our decision-making for this specific branch point, we did not perform this pairwise comparison for all classification distinctions, which is an area of future research.

## Conclusion

We present here a validated and reliable approach for functional UE motion classification in humans. The taxonomy enables the parsing of activities and functional movements into overarching motion characteristics and fundamental units of measure. The taxonomy can thus be expected to serve both clinical and research applications. It can objectively quantify rehabilitation training dose, enabling dose-response investigations in varied clinical contexts. It also provides a codified terminology, ensuring confidence that knowledge from the study of functional motion can be appropriately pooled. If we want to improve clinical and mechanistic insights into motor recovery and control, our methodological arsenal stands to benefit from a systematic nomenclature.

## Data Availability

The datasets generated for this study are available on request to the corresponding author.

## Ethics Statement

This study was carried out in accordance with the recommendations of Declaration of Helsinki. The protocol was approved by the NYU Institutional Review Board.

## Author Contributions

All authors contributed to the creation and refinement of the taxonomy. HS, AP, and DN designed the tasks and wrote the manuscript. AP and NP collected and coded the data. HS and AP performed the analyses.

### Conflict of Interest Statement

The authors declare that the research was conducted in the absence of any commercial or financial relationships that could be construed as a potential conflict of interest.

## Supplementary Material

Video S1 Drinking (part of eating activity).

Video S2 Applying deodorant (part of self-hygiene).

Video S3 Rolling up sleeve (part of dressing).
